# Supplementary material for: The genome sequence of a fungus weevil, Pseudeuparius sepicola (Fabricius, 1792)
Source: Wellcome Open Res. 2024 Oct 18;9:615. [Version 1] doi: 10.12688/wellcomeopenres.23209.1 (PMC11576567; doi:10.12688/wellcomeopenres.23209.1)
Supplement: Peer review history

# Reviewer report: Reviewer response for version 1

**DOI:** 10.21956/wellcomeopenres.25561.r107617

**Published:** 2024-11-19

**Authors:** Elina Immonen

**Affiliations:** 1 Uppsala University, Uppsala, Sweden

In this Data note the authors have sequenced and assembled the nuclear and mitochondrial genome of a fungus weevil, *Pseudeuparius sepicola, *male, using PacBio Hi-Fi reads and Hi-C data. The methods are appropriate and the assembly quality is good. The nuclear genome assembly is scaffolded at a chromosome level, including the ten autosomes and the X and Y sex chromosomes, with the N50 score of 68.5 Mb and BUSCO score of completeness of 98.5%. The authors have conducted some manual curation of scaffolds, which has helped to join some scaffolds and reduce their total number to the chromosome level. The final assembly has a total length of 769.1Mb. The sex chromosomes were identified based on read coverage statistics, but no more information is provided. It is not stated how complete the sex chromosomes are likely to be. The genome is not yet annotated, and once done, this data will be even more important addition to the increasing number of Coleopteran genomes.

Are sufficient details of methods and materials provided to allow replication by others?

Yes

Is the rationale for creating the dataset(s) clearly described?

Yes

Are the datasets clearly presented in a useable and accessible format?

Yes

Are the protocols appropriate and is the work technically sound?

Yes

Reviewer Expertise:

Evolutionary genomics using multi-omic approaches.

I confirm that I have read this submission and believe that I have an appropriate level of expertise to confirm that it is of an acceptable scientific standard.

# Reviewer report: Reviewer response for version 1

**DOI:** 10.21956/wellcomeopenres.25561.r107620

**Published:** 2024-10-30

**Authors:** Bhagya C Thimmappa

**Affiliations:** 1 USDA, USDA Agricultural Research Service, Chatsworth, NEW JERSEY, USA

The manuscript titled "The genome sequence of a fungus weevil, *Pseudeuparius sepicola"* by Booth et al. reports a nuclear genome and mitochondrial assembly of the fungus weevil, *Pseudeuparius sepicola. *Pacific Biosciences single molecule HiFi long reads and chromosome conformation Hi-C data were used to ensure comprehensive assembly with chromosomal-level scaffolding and an N50 of 68.5 Mb. The genome assembly is nearly complete, with a BUSCO score of 98.5%. The methodology is well documented, and additional manual curation leads to reliable data to study the evolutionary biology *and *genetics *of Pseudeuparius sepicola* and related species in the future.

 The genome and mitochondrial assembly reported in this work significantly contribute to the genomic resources available for this species. However, additional genome and mitochondrial annotation could further enhance this dataset's impact, along with more discussion on how the genome size compares to other closely related species.

Are sufficient details of methods and materials provided to allow replication by others?

Yes

Is the rationale for creating the dataset(s) clearly described?

Yes

Are the datasets clearly presented in a useable and accessible format?

Yes

Are the protocols appropriate and is the work technically sound?

Yes

Reviewer Expertise:

Bioinformatics, Genomics, Multi-Omics

I confirm that I have read this submission and believe that I have an appropriate level of expertise to confirm that it is of an acceptable scientific standard.
